# Investigation of energy band at atomic layer deposited AZO/β-Ga_2_O_3_ ($\overline{2}01$) heterojunctions

**DOI:** 10.1186/s11671-019-3092-x

**Published:** 2019-08-14

**Authors:** Shun-Ming Sun, Wen-Jun Liu, Dmitriy Anatolyevich Golosov, Chen-Jie Gu, Shi-Jin Ding

**Affiliations:** 1 State Key Laboratory of ASIC and System, School of Microelectronics, Fudan University, Shanghai, 200433 China; 2 Belarusian State University of Informatics and Radioelectronics, P. Brovka Street 6, 220013 Minsk, Belarus; 3 Division of Microelectronics, School of Science, Ningbo University, Ningbo, 315211 China

**Keywords:** β-Ga_2_O_3_, Contacts, Intermediate semiconductor layer

## Abstract

The Al-doped effects on the band offsets of ZnO/β-Ga_2_O_3_ interfaces are characterized by X-ray photoelectron spectroscopy and calculated by first-principle simulations. The conduction band offsets vary from 1.39 to 1.67 eV, the valence band offsets reduce from 0.06 to − 0.42 eV, exhibiting an almost linear dependence with respect to the Al doping ratio varying from 0 to 10%. Consequently, a type-I band alignment forms at the interface of ZnO/β-Ga_2_O_3_ heterojunction and the AZO/β-Ga_2_O_3_ interface has a type-II band alignment. This is because incorporating Al into the ZnO would open up the band gaps due to the strong Al and O electron mixing, and the conduction and valence band edges consequently shift toward the lower level.

## Background

Recently, an oxide semiconductor Ga_2_O_3_ has attracted widespread interests because of its unique characteristics such as the large bandgap, high saturation electron velocity, and high temperature resistance [1]. There are five kinds of isomers for Ga_2_O_3_: α, β, γ, δ, and ε, where β-Ga_2_O_3_ can be grown easier and has been studied widely [2]. In particular, β-Ga_2_O_3_ has a larger breakdown electric field than that of traditional third-generation semiconductor materials, such as SiC and GaN [3]. The n-type conductive properties can be modulated by doping Sn [4] or Si [5]. So β-Ga_2_O_3_-based devices [6, 7] have broad application prospects in the fields of information technology, energy conservation, and emission reduction. However, β-Ga_2_O_3_-based devices have a common limitation: the contact between β-Ga_2_O_3_ and most metals tends to be Schottky because of the large barrier induced by the wide bandgap and finite carrier concentration. In recent years, inserting an interlayer, such as ITO [8] and AZO [9], between Ga_2_O_3_ and metals is shown to be a valid method to reduce the energy barrier between β-Ga_2_O_3_ and metal.

Al-doped zinc oxide (ZnO) has gained much attention because of low resistivity and lower fabrication cost than ITO [10]. In particular, the high thermal stability, high mobility, and carrier concentration make it a promising candidate of the intermediate semiconductor layer (ISL) [11]. So far, Al-doped ZnO films can be grown through the following techniques: molecular beam epitaxy (MBE) [12], magnetron sputtering [13], chemical vapor deposition (CVD) [14], and atomic layer deposition (ALD) [15]. Specially, ALD is a renowned method to prepare nano-thickness film which exhibits large area excellent uniformity and unites growth rate per cycle because of the self-limiting surface reaction including the self-limiting chemical adsorption and self-limiting sequential reaction [16]. Moreover, ALD can reduce interface disorder and more precise modulate the Al doping concentration by changing the ratios of growth cycles.

Note that the conduction band offset (CBO) determines the energy barrier for the electron transport, so a smaller CBO is beneficial to form an Ohmic contact. Based on our previous work [17], by increasing Al doping concentration, the Al-doped ZnO film changes from polycrystalline to amorphous nature, and its bandgap widens as well. However, the band offsets of different Al-doped ZnO/β-Ga_2_O_3_ heterojunctions have not been studied widely. In this work, the ZnO films with different Al doping ratios were respectively deposited on β-Ga_2_O_3_ substrates by ALD. The results show the VBO and CBO are almost linearly dependent on the Al doping ratio.

## Methods

The substrates are bulk β-Ga_2_O_3_ ($\overline{2}01$) and the doping concentration is about 3 × 10^18^/cm^3^. The cleaning process for Ga_2_O_3_ substrates was undergone ultrasonic wash in acetone and isopropanol for each 10 min with repeated three times. Subsequently, the Ga_2_O_3_ substrates were rinsed with deionized water. Afterwards, the Al-doped ZnO films were grown onto the Ga_2_O_3_ substrate by ALD (Wuxi MNT Micro Nanotech Co., LTD, China). Three kinds of samples were prepared. Firstly, the undoped ZnO films were grown by ALD with the precursors of Zn (C_2_H_5_)_2_ (DEZ) and H_2_O at 200 ^o^C. Secondly, the Al-doped ZnO films were carried out by adding one pulse of trimethylaluminum (TMA) and H_2_O every 19th cycle of DEZ and H_2_O pulsing (denoted as 5% Al doping) at a substrate temperature of 200 ^o^C during ALD. Thirdly, the Al-doped ZnO films of ratio 9:1 (denoted as 10% Al doping) were also prepared. The growth rate of ZnO and Al_2_O_3_ was 0.16 and 0.1 nm/cycle, respectively. Every kind film included two different thicknesses, i.e., 40 nm and 10 nm for the thick and thin film, respectively. In addition, the β-Ga_2_O_3_ substrate was used to study the bulk material. Ga 2*p*, Zn 2*p* CLs, and the valence band maximum (VBM) were measured by X-ray spectroscopy (XPS) (AXIS Ultra DLD, Shimadzu) and the step of resolution XPS spectra is 0.05 eV. To avoid the surface contamination of the sample during the transfer process from ALD to XPS chamber, Ar ion etching was performed before the XPS measurement. Note that the charging effect can shift the XPS spectrum, and the BE of C 1*s* peak is calibrated at 284.8 eV to solve the problem.

## Results and Discussions

The valence band offset (VBO) of Al-doped ZnO/β-Ga_2_O_3_ heterojunction can be obtained through the formula as follows [18]:

1 $$ \Delta  {E}_V=\left({E}_{\mathrm{Ga}\ 2p}^{{\mathrm{Ga}}_2{\mathrm{O}}_3}-{E}_{\mathrm{VBM}}^{{\mathrm{Ga}}_2{\mathrm{O}}_3}\right)-\left({E}_{\mathrm{Zn}\ 2p}^{\mathrm{AZO}}-{E}_{\mathrm{VBM}}^{\mathrm{AZO}}\right)-\left({E}_{\mathrm{Ga}\ 2p}^{{\mathrm{Ga}}_2{\mathrm{O}}_3}-{E}_{\mathrm{Zn}\ 2p}^{\mathrm{AZO}}\right) $$

where${E}_{\mathrm{Ga}\ 2p}^{{\mathrm{Ga}}_2{\mathrm{O}}_3}$ refers to the binding energy (BE) of Ga 2*p* core level (CL) in bulk β-Ga_2_O_3_, ${E}_{\mathrm{VBM}}^{{\mathrm{Ga}}_2{\mathrm{O}}_3}$ refers to the BE of VBM in bulk β-Ga_2_O_3_, ${E}_{\mathrm{Zn}\ 2p}^{\mathrm{AZO}}$ refers to the BE of Zn 2*p* CL in thick Al-doped ZnO films, ${E}_{\mathrm{VBM}}^{\mathrm{AZO}}$ refers to the BE of VBM in thick Al-doped ZnO films. The latter ${E}_{\mathrm{Ga}\ 2p}^{{\mathrm{Ga}}_2{\mathrm{O}}_3}$ and ${E}_{\mathrm{Zn}\ 2p}^{\mathrm{AZO}}$ refer to the BE of Ga 2*p* and Zn 2*p* CLs in thin Al-doped ZnO films, respectively.

Subsequently, based on the *E*_*g*_ and *∆E*_*V*_, the CBO at the Al-doped ZnO/β-Ga_2_O_3_ interface can be calculated by the following equation:

2 $$ \Delta  {E}_C={E}_g^{{\mathrm{Ga}}_2{\mathrm{O}}_3}-{E}_g^{\mathrm{AZO}}-\Delta  {E}_V $$

where${E}_g^{{\mathrm{Ga}}_2{\mathrm{O}}_3}$ is the bandgap of Ga_2_O_3_ and ${E}_g^{\mathrm{AZO}}$ is the bandgap of Al-doped ZnO. The bandgaps for undoped, 5% Al-doped ZnO, 10% Al-doped ZnO, and β-Ga_2_O_3_ are 3.20 eV, 3.25 eV, 3.40 eV, and 4.65 eV, respectively [17, 19]. The bandgap increases with a higher Al doping ratio, agreeing well with the simulation in the next part.

**Fig. 1:**
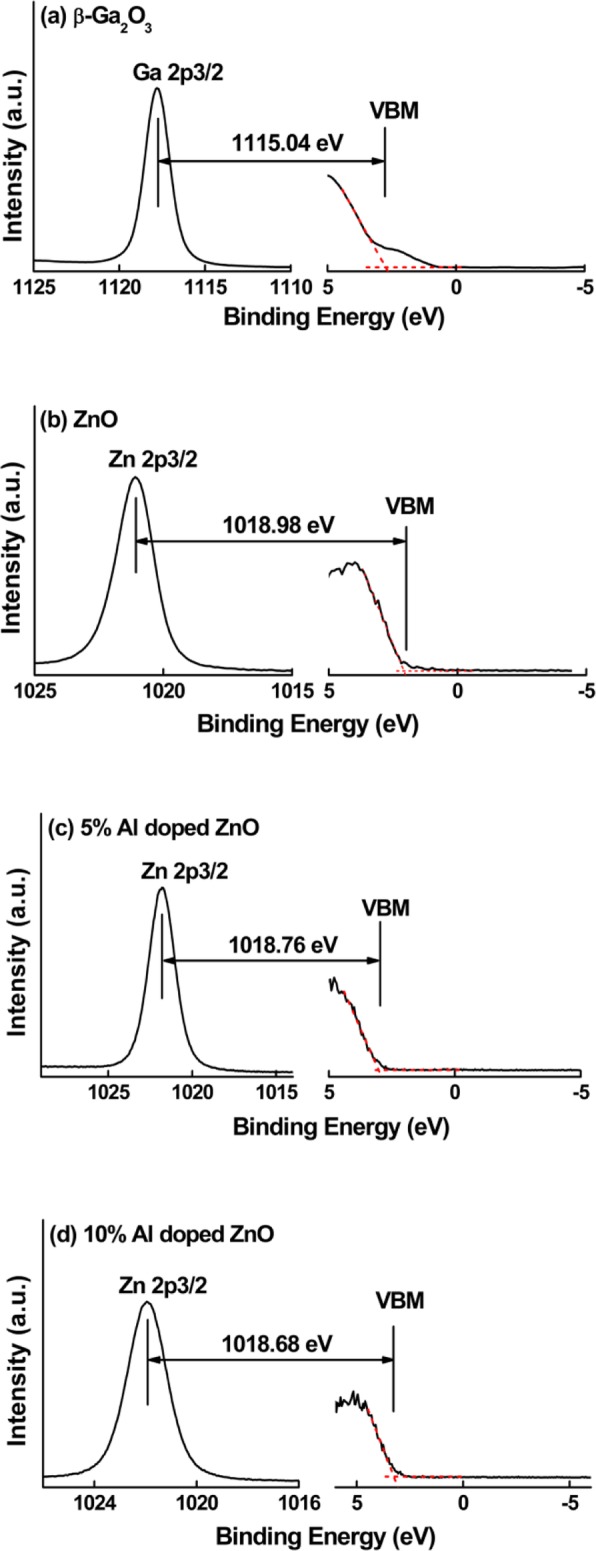
High resolution XPS spectra for core level and valence band maximum (VBM) of **a** Ga 2*p* core level spectrum and VBM from bare β-Ga_2_O_3_, **b** Zn 2*p* core level spectrum and VBM from thick pure ZnO/β-Ga_2_O_3_, **c** Zn 2*p* core level spectrum and VBM from thick 5% Al-doped ZnO/β-Ga_2_O_3_, and **d** Zn 2*p* core level spectrum and VBM from thick 10% Al-doped ZnO/β-Ga_2_O_3_

**Fig. 2:**
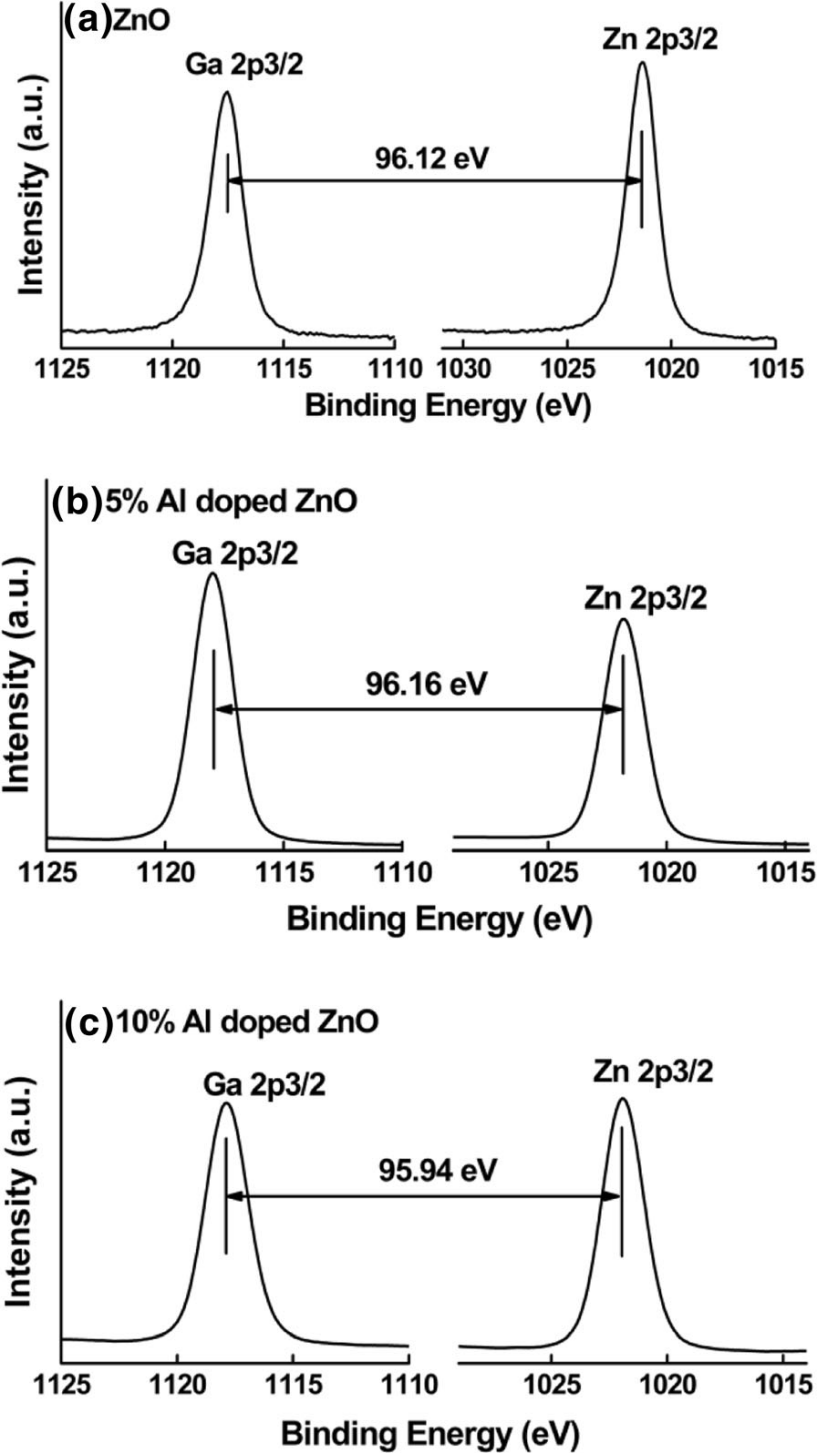
The core level spectra of Ga 2*p* and Zn 2*p* obtained from high resolution XPS spectra of **a** thin ZnO/β-Ga_2_O_3_, **b** thin 5% Al-doped ZnO/β-Ga_2_O_3_, and **c** thin 10% Al-doped ZnO/β-Ga_2_O_3_

Figure 1 shows the Ga and Zn element CLs and VBM of bulk β-Ga_2_O_3_, thick undoped, and 5% and 10% Al-doped ZnO films. Fitting the linear area and the flat band zone from the VBM spectrum can deduce the VBM [20]. Figure 2 shows Ga 2*p* and Zn 2*p* CL from various thin Al-doped ZnO/β-Ga_2_O_3_ heterojunctions. The BE differences of Ga 2*p* and Zn 2*p* CLs for the undoped, 5% Al-doped ZnO/β-Ga_2_O_3_, and 10% Al-doped ZnO/β-Ga_2_O_3_ are obtained to be 96.12 eV, 96.16 eV, and 95.94 eV, respectively. Then, the VBOs at the interfaces are determined to be 1.39 eV, 1.52 eV, and 1.67 eV for the undoped, 5% Al-doped ZnO/β-Ga_2_O_3_, and 10% Al-doped ZnO/β-Ga_2_O_3_ samples, respectively.

**Fig. 3:**
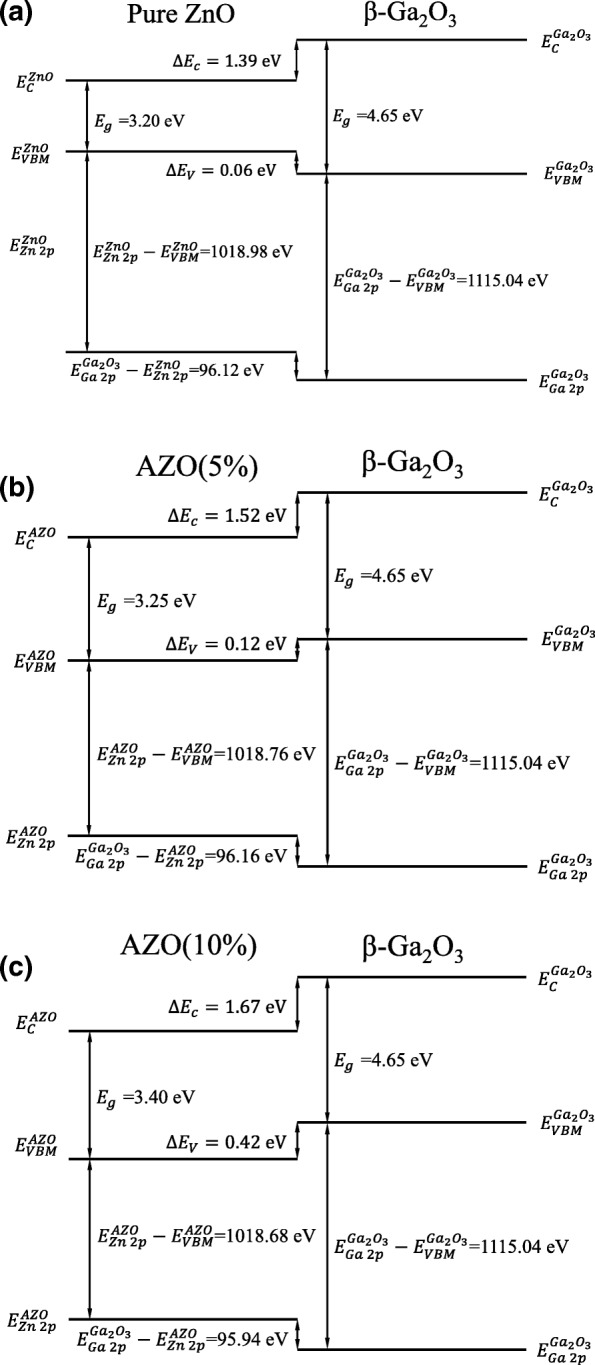
Schematic band alignment diagram of **a** pure ZnO/β-Ga_2_O_3_, **b** 5% Al-doped ZnO/β-Ga_2_O_3_, and **c** 10% Al-doped ZnO/β-Ga_2_O_3_

**Fig. 4:**
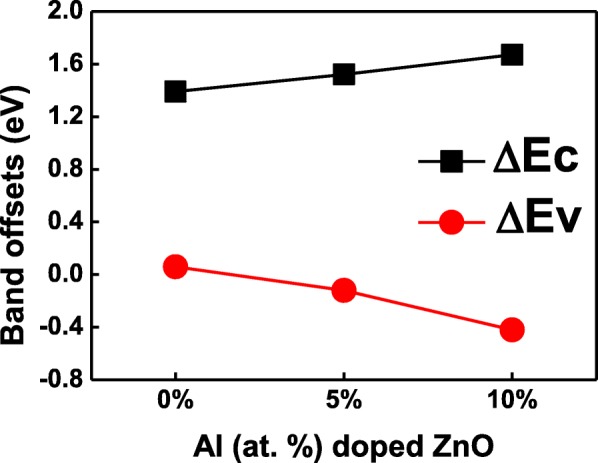
The conduction and valence band offsets of atomic-layer-deposited AZO/β-Ga_2_O_3_ heterojunctions fabricated at different Al doping ratios

The systematic band alignment for the 0%, 5%, and 10% Al-doped ZnO/β-Ga_2_O_3_ heterojunctions are calculated by the above equations, as shown in Fig. 3. The band offset of undoped ZnO/β-Ga_2_O_3_ heterojunction belongs to type I. While both 5% and 10% Al-doped ZnO/β-Ga_2_O_3_ heterojunctions have type-II band offsets. Figure 4 depicts the band alignments of Al-doped ZnO/β-Ga_2_O_3_ interfaces have a similar linear relationship with Al doping concentration. The CBO varies from 1.39 to 1.67 eV with the Al-doped concentration increasing from 0 to 10%. While the VBO reduces from 0.06 to − 0.42 eV with the Al-doped concentration rising from 0 to 10%. It is noted that the CBO and VBO for sputtered AZO/β-Ga_2_O_3_ are 0.79 eV and 0.61 eV, respectively [9]. Both the conduction and valence band shift downward in this work, which could be due to the different composition ratio and crystalline structure introduced by deposited methods.

Other than that, first-principle simulations were performed by the Vienna Ab-initio Simulation Package (VASP) [21–24] to investigate the electronic band structure and band alignment of Al-doped ZnO/Ga_2_O_3_ heterojunctions. During the calculation, the electron-ion interactions were treated by the ultra-soft pseudo-potentials, and the wave functions and potentials were expanded by the plane-wave basis [25]. Besides, generalized gradient approximation (GGA) proposed by Perdew, Burke, and Ernzerhof (PBE) was implemented to describe the exchange-correlation energies [26]. Prior to initiating the simulation, converging tests were performed. It showed that the cutoff energy of 450 eV for the plane-wave basis and k-space grids of 3 × 3 × 3 with the Monkhorst Pack scheme gave the well-converged results. In the structure optimization, a conjugate gradient method was used and the residual force was released until it was less than 0.01 eV/Å. Moreover, the hybrid density functions based on the semi-local PBE approximation were implemented. To correct the underestimated bandgap, 35% of PBE exchange was replaced with the exact one [27]. To identify the band edge shift with the change of the Al doping level, the average electrostatic potential (AEP) was calculated and aligned to the vacuum level which was scaled to 0 V. The VBM and conduction band minimum (CBM) were consequently aligned to the AEP based on the band diagram [28]. In this work, bulk ZnO with 16 O atoms and 16 Zn atoms in the supercell was used. To introduce the Al doping, one or two Zn atoms in the supercell were replaced by the Al atoms, creating the Al-doped structure with the doping concentration of 3.21% and 6.25 %, respectively.

**Fig. 5:**
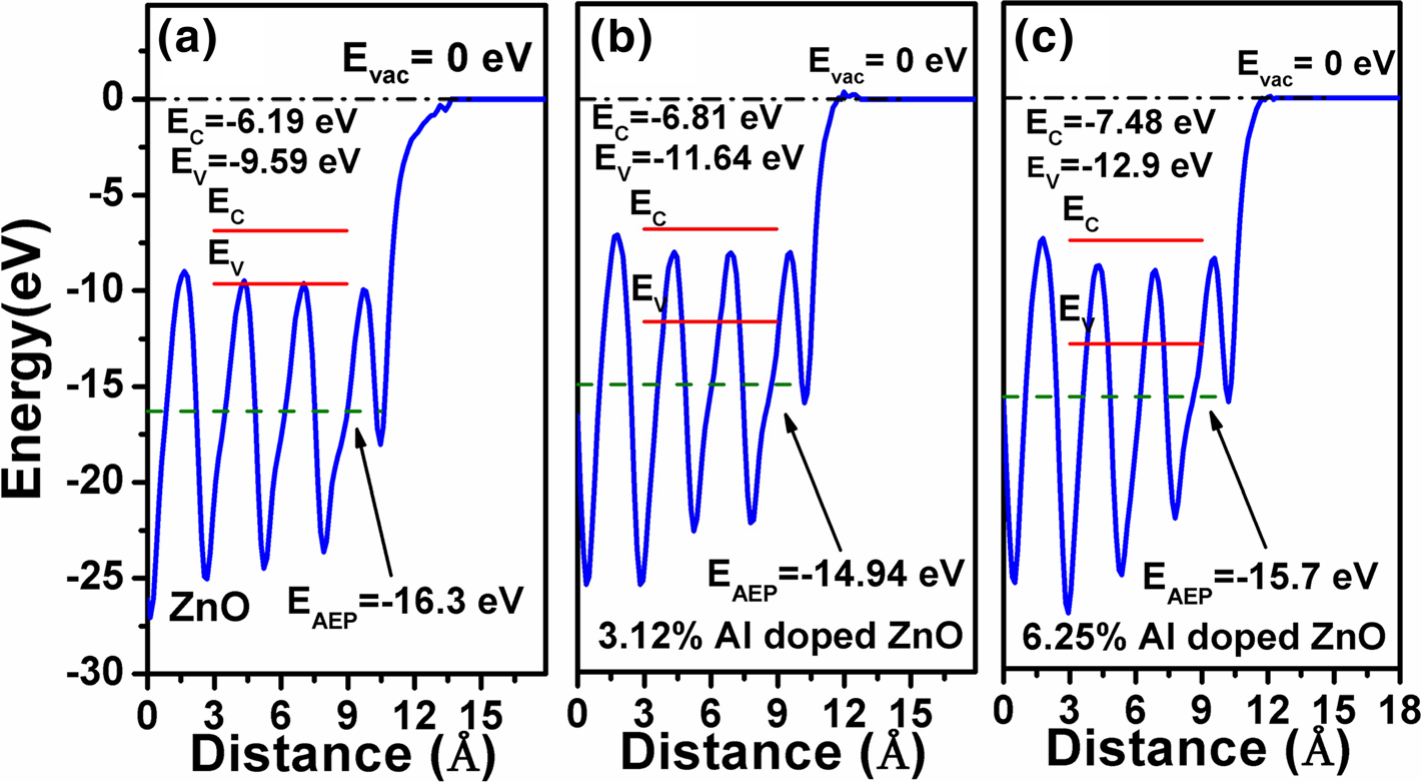
The calculated band diagram of **a** undoped ZnO, **b** 3.21% Al-doped ZnO, and **c** 6.25% Al-doped ZnO structure. The Fermi levels were set to 0 eV

Figure 5 a–c shows the calculated band diagrams of the undoped, 3.21% Al-doped ZnO, and 6.25% Al-doped ZnO structures, respectively. It clearly shows that ZnO is a direct bandgap semiconductor with the bandgap of 3.42 eV, and the CBM as well as the VBM was located at the Γ point of Brillouin zone. These theoretical simulation results match the experimental value quite well [29]. With the Al doping, it could be found that the Fermi levels shifted upwards into the conduction band, which converts the pure ZnO into an n-type semiconductor. In the meanwhile, the bandgaps also increased to 4.83 eV and 5.42 eV for 3.21% Al-doped ZnO and 6.25% Al-doped ZnO, respectively. Although the bandgaps here for the doped ZnO are higher than our experimental results; however, this could be ascribed to the neglecting of interfacial defect states as well as other crystal defects.

**Fig. 6:**
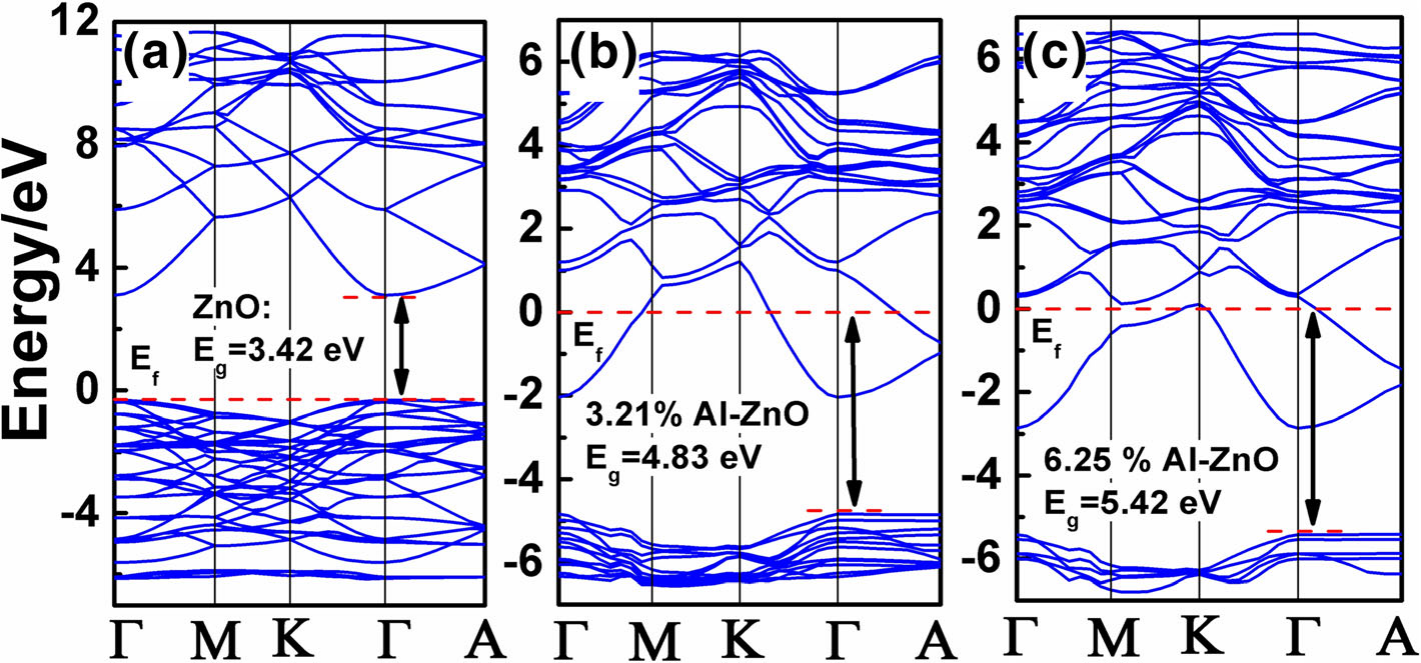
The band alignment of AZO/β-Ga_2_O_3_ heterojunctions with **a** undoped, **b** 3.21%, and **c** 6.25% Al-doped ZnO. The vacuum levels were scaled to 0 eV

Figure 6 a–c presents the band alignments of undoped, 3.21% Al-doped ZnO, and 6.25% Al-doped ZnO to the vacuum level. For the conduction bands of the materials, due to the strong electron mixing between the Al and O element, it could be found that the energy level decreases from − 6.19 eV of the ZnO to − 6.81 eV for the 3.21% Al-doped ZnO (Δ*E* = 0.62 eV ) and further decreases to − 7.48 eV for the 6.25% Al-doped ZnO (*ΔE* = 1.29 eV ). In the meanwhile, due to the opening up of the bandgap, it also could be found that the valence band edge moves downwards from − 9.59 eV for the ZnO to − 11.64 eV for 3.21% Al-doped ZnO (*ΔE* = 2.05 eV ) and − 12.9 eV for the 6.25% Al-doped ZnO (*ΔE* = 3.31 eV ). In all, ascribed to the strong Al and O electron mixing, it could be understood that incorporating Al in the ZnO would open up the band gaps. Moreover, it would shift both the conduction band and valence band edge towards the lower energy level when aligned to the vacuum level.

## Conclusions

In conclusion, the band alignments of different Al-doped ZnO/β-Ga_2_O_3_($\overline{2}$01) interfaces have been investigated by XPS. A type-I band alignment forms at the interface of ZnO/β-Ga_2_O_3_ heterojunction. While the AZO/β-Ga_2_O_3_ interface has a type-II band alignment. The CBOs vary from 1.39 to 1.67 eV and the VBOs reduce from 0.06 to − 0.42 eV with the Al-doped concentration rising from 0 to 10%. Moreover, the density function calculations show that band offsets change due to strong Al and O electron mixing when Al is incorporated into ZnO. These results suggest that the pure ZnO is a valid ISL to reduce the barrier height and promote the electron transport.

## Data Availability

The datasets supporting the conclusions of this manuscript are included within the manuscript.

## Abbreviations

AEP: Average electrostatic potential
ALD: Atomic layer deposition
BE: Binding energy
CBM: Conduction band minimum
CBO: Conduction band offset
CL: Core level
CLs: Core levels
CVD: Chemical vapor deposition
DEZ: Zn (C_2_H_5_)_2_
Ga_2_O_3_: Gallium oxide
GaN: Gallium nitride
GGA: Generalized gradient approximation
ISL: Intermediate semiconductor layer
PBE: Perdew, Burke, and Ernzerhof
SiC: Silicon carbide
TMA: Trimethyaluminum
VASP: Vienna Ab initio Simulation Package
VBM: Valence band maximum
VBO: Valence band offset
XPS: X-ray spectroscopy
ZnO: Zinc oxide
